# Use of antipsychotic medication with weight-inducing potential in first-episode psychosis

**DOI:** 10.3389/fpsyt.2026.1828874

**Published:** 2026-07-24

**Authors:** Maren Johanne Landsem, Gunnar Morken, Morten Brix Schou

**Affiliations:** 1 Faculty of Medicine and Health Sciences, Norwegian University of Science and Technology, NTNU, Trondheim, Norway; 2 Department of Psychiatry, St Olavs University Hospital, Trondheim, Norway; 3 Department of Psychosis and Rehabilitation, St Olavs University Hospital, Trondheim, Norway

**Keywords:** acute psychosis, antipsychotic treatment, first-episode psychosis, psychosis treatment, schizophrenia, weight gain

## Abstract

**Background:**

Antipsychotic medication is recommended in the treatment of first-episode psychosis (FEP). Modern international guidelines do not recommend using antipsychotic medication with a high risk of weight-inducing potential (HWPA) as the first choice. However, many studies find the use of such antipsychotic medication in rates ranging from 30% to 60% among FEP patients, including in Norway. This study investigated the rates of HWPA use in FEP patients and possible demographic and clinical factors associated with its use.

**Methods:**

This was a retrospective cohort study including all FEP patients treated at St. Olavs University Hospital from 2012 to 2016. Data regarding antipsychotic medication, demographic, and clinical variables were collected from the electronic records of all patients at treatment initiation and on use of medication at 1- and 2-year follow-up. Antipsychotic medications were categorized as HWPA or low/medium risk of weight-inducing potential (L/MWPA).

**Results:**

In total, 238 patients were included, of whom 123 (52%) were given HWPA as first medication, 83 patients (35%) were given L/MWPA, while 32 patients (13%) received no antipsychotic medication at treatment initiation. Higher age, acute referral, and severity of illness were associated with a higher risk of treatment with HWPA.

**Conclusion:**

A large proportion of FEP patients in this cohort were initiated on HWPA as the first-choice medication. To ensure adherence to guidelines, there must be a focus on reducing the use of HWPA in FEP patients in general and in groups with a higher risk of being initiated on these antipsychotic medications.

## Introduction

The debut of a psychotic condition often has severe consequences for patients and their families. Nonaffective psychotic disorders have a lifetime prevalence of 1% to 2% (1). Most patients experiencing a first episode of psychosis (FEP) are recommended treatment with antipsychotic medication, as these medications are effective in reducing symptoms in the acute phase and in reducing the risk of relapse in patients with chronic psychotic disorders (2, 3).

Since antipsychotic treatment often spans longer time periods, from months to years, the wide range of side effects associated with antipsychotic medications (4) can present a significant issue for the patients’ health and recovery.

Weight gain is a severe side effect of some second-generation antipsychotics (SGA), and service users rate weight gain as the most distressing side effect of antipsychotic treatment (5). Weight gain is also recognized as an important reason for nonadherence to antipsychotic treatment (6).

Antipsychotics can be categorized according to the likelihood of weight gain as a side effect. A high risk is seen with olanzapine, clozapine, and chlorpromazine, while other antipsychotics are categorized as medium or low risk (7, 8). Olanzapine is a SGA that, in particular, causes a significant increase in body weight (9, 10). In a large meta-analysis, the mean weight gain was 7.5 kg for patients with first-episode psychosis treated with olanzapine, and longer study duration was associated with greater weight gain. For studies with 13 weeks or more of follow-up, mean weight gain was 11.4 kg, indicating a link between treatment length and severity of weight gain as an adverse effect (11). Other meta-analyses also confirm olanzapine’s weight-inducing potential (12, 13).

Guidelines from both Scandinavia and other countries over the last 15 years (3, 14–17) recommend not using olanzapine as the first or second choice of antipsychotic medication for patients with FEP due to side effects such as weight gain. However, the clinical use of antipsychotics with a high risk of weight-inducing potential (referred to in this article as HWPA) has been shown in studies to vary from 13% to 80% (18–23) in patients with FEP. Norwegian guidelines did not, until an update in 2025, take this issue into account; they now state that “for FEP patients, one should use a SGA with a low risk of unfortunate metabolic side effects and weight gain” (24). In studies from Norway, the use of HWPA is 42%–47% in patients with FEP (25, 26), making it by far the most used antipsychotic medication in FEP treatment, and not in line with the new recommendations.

Therefore, as guidelines are moving away from HWPA as the first-choice medication in patients with FEP, it is important to gain more knowledge about the prescription practices of these medications. Such knowledge can help clinicians adhere to guidelines associated with fewer severe side effects, improved adherence to medication, and thus possibly a better quality of life for patients with FEP. The primary aim of this study is to describe the use of antipsychotic medication with different weight-inducing potentials at illness presentation among FEP patients in a hospital catchment area. The study also aims to identify possible demographic and clinical predictors of HWPA use in patients with FEP and to describe the use of different weight-inducing groups of antipsychotics during the first 2 years of treatment.

## Materials and methods

### Study design and participants

This is a retrospective naturalistic cohort study, which is part of a larger quality assurance project regarding the treatment for patients with FEP at St. Olavs University Hospital, Trondheim, Norway. The participants have received treatment at St. Olavs University Hospital in the catchment area of Sør-Trøndelag county, which had 313,370 inhabitants in 2016 (27). Patients presenting to any healthcare services with symptoms of FEP are referred to St. Olavs University Hospital for diagnostics and treatment, which is the only hospital providing treatment for psychotic disorders in the catchment area. The patients are evaluated by a group of clinicians consisting of psychiatrists, psychologists, and nurses. The data included all patients with a registered hospital contact between 01 January 2012 and 31 December 2016, where the patients received an International Classification of Diseases, Tenth Revision (ICD-10) diagnosis of schizophrenia (F20.x), persistent delusional disorders (F22.x), acute and transient psychotic disorders (F23.x), induced delusional disorder (F24.x), schizoaffective disorder (F25.x), other psychotic disorder not due to a substance or known physiological condition (F28), or unspecified nonorganic psychosis (F29) for the first time in their life. Electronic patient records were thoroughly assessed to exclude patients with a FEP diagnosis prior to 01 January 2012. The inclusion criteria for the study were age 18 to 50 years at first contact with specialized mental healthcare leading to a psychosis diagnosis [ICD-10 F.20.x–F29, apart from schizotypal disorder (F21)]. The upper age limit of 50 years is set because nonaffective psychosis primarily debuts before age 50 (28). The data included spans the preceding 2 years until patients moved out of the study’s catchment area or died.

### Study variables

The prescribed type of antipsychotic medication was registered at treatment initiation, after 1 and 2 years of treatment. Antipsychotic medication was categorized as HWPA or low/medium risk of weight-inducing potential (L/MWPA) according to two studies categorizing the side effects of antipsychotic medications (7, 8). Clozapine is categorized as HWPA (7, 8). However, due to its special role in the treatment of treatment-resistant schizophrenia (29), the use of clozapine was analyzed separately when describing antipsychotic medication use at the 1- and 2-year follow-up.

Demographic variables such as age, gender, education, and employment status at referral were collected from the electronic patient records. Education was categorized based on the highest level of education completed at referral to specialist mental healthcare in three categories: elementary school (9 years of education), high school (12 years of education), and college or university (3 years or more of college or university education). Employment status at the time of referral was categorized into three groups: in school or studies, employed, or not employed or in education.

Clinical variables possibly affecting the choice of medication, and reliably recorded in the electronic patient records, were collected from the electronic patient records. These variables included current substance use at referral, categorized as present or not present, and whether patients were referred for acute or elective treatment. As a proxy for the severity of the disorder, the level of care at referral was used. It was categorized into three levels (outpatient care, voluntary inpatient care, and involuntary inpatient care), as studies have shown that both symptom severity and functioning are associated with voluntary vs. involuntary treatment (30, 31). The psychosis diagnoses were categorized into four groups: schizophrenia/schizoaffective disorder (F20.x and F25.x), persistent paranoid psychosis (F22.x), acute and transient psychosis (F23.x), and unspecified nonorganic psychosis (F29), as subgroups of psychosis. The diagnoses were obtained from electronic patient records. The diagnosis used in this article was the final diagnosis in the ICD-10 F20 chapter set during the follow-up period of 2 years, although other psychosis diagnoses may have been used temporarily during this period.

When describing the use of antipsychotic medication at 1- and 2-year follow-up, acute and chronic psychotic disorders were separated, as the former group is usually not eligible for antipsychotic treatment beyond 1 year. A chronic psychotic disorder is defined, in this paper, as a final diagnosis of F.20x, F22.x, F25.x, and F29, while an acute psychotic disorder is defined as a final diagnosis of F23.x. Missing antipsychotic status is divided into two categories: missing due to no longer being followed up by specialist healthcare (while still living in the catchment area), or missing due to having moved out of the catchment area or having died.

### Statistical methods

Demographic and clinical data are described across groups of patients using HWPA medication, L/MWPA, and no antipsychotic medication, using medians, interquartile ranges, and proportions. Kruskal–Wallis test was used to examine differences in continuous variables, as these were not normally distributed, with *post hoc* testing between groups using Dunn’s test with Bonferroni correction for multiple testing. The Chi-square test was used to examine differences in proportions of categorical data. Furthermore, adjusted standardized residuals were used to evaluate which groups and their proportions contributed to statistically significant findings (32). Here, we report groups with *p*-values < 0.05 and those reaching statistical significance after Bonferroni correction for multiple testing. The statistical significance level was set at *p* < 0.05 or was adjusted for multiple testing where appropriate. To describe the use of the different groups of weight-inducing potential antipsychotic medication at initiation and during the 1- and 2-year follow-up, we used Sankey diagrams, created using https://sankeymatic.com/. IBM SPSS Statistics version 30 was used for statistical calculations.

## Results

### Study population

**Figure 1:**
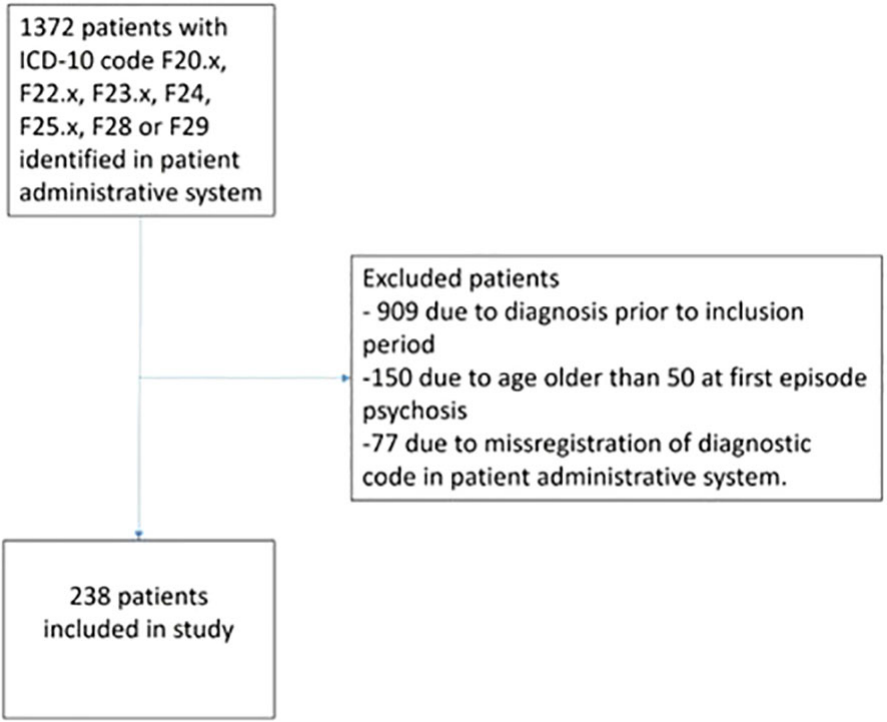
Reasons for exclusion of patients identified in the patient administrative system with an F20–F29 ICD-10 diagnosis.

A total of 1,372 patients with a psychosis diagnosis were identified in the patient administrative system during the inclusion period. Of these, 238 patients with FEP were included. Excluded patients were 909 who were diagnosed prior to 01 January 2012, 148 who were older than 50 years, and 77 with misregistered diagnostic codes in the patient administrative system. See **Figure 1** for patient flow. The median age of the included patients was 28 (IQR: 23–36) years, with a higher median age among women compared with men, 30 years vs. 26 years, respectively. Of the included patients, 156 were men and 82 were women. Almost two-thirds of the population were not in work or school activity at referral. Approximately one-third of the patients had active use of alcohol or other substances at referral. Slightly more than 70% of patients were acutely referred, and the same proportion were treated as inpatients at their first contact, leading to the psychosis diagnosis. **Table 1** provides an overview of demographic and clinical variables divided into participants using HWPA and L/MWPA. Antipsychotic medication at initiation was collected for all 238 patients, whereas at the 1- and 2-year follow-up, there were missing data on antipsychotic medication use for 73 (31% of the cohort) and 102 (43% of the cohort) patients, respectively. Missing data occurred because patients did not have any follow-up in specialist mental healthcare, had moved out of the catchment area, or had died (*n* = 2). For patients with chronic psychotic disorders, the proportion of missing data was 24% and 38% at the 1- and 2-year follow-up, respectively.

**Table 1:** Demographic and clinical characteristics of participants using high or low/medium weight-inducing potential antipsychotics as the first prescribed antipsychotic medication.

	Groups of antipsychotic medications			Total population (*n* = 238)	Statistical testing
Antipsychotic weight-inducing potential	High (*n* = 123; 52%)	Low/medium (*n* = 83; 35%)	No use (*n* = 32; 13%)		
Antipsychotic medication	Olanzapine (*n* = 123)	Quetiapine (*n* = 33)			
		Aripiprazole (*n* = 31)			
		Risperidone/paliperidone (*n* = 7)			
		Perphenazine (*n* = 5)			
		Other (*n* = 7)^†^			
Age (median; IQR)	29.0 (23; 38)^‡^	26.0 (22; 31)^‡^	29.5 (24; 36)	28.0 (23; 36)	*Z* = 6.93; *p* = 0.03
Gender (*n*; %)					
Men	84 (54)	50 (32)	22 (14)	156 (66)	*χ*^2^ = 1.59; *p* = 0.45
Women	39 (48)	33 (40)	10 (12)	82 (34)	
Education (*n*; %) (*n* = 214)					
≤ 9 years of education	37 (43)	39 (45)	11 (13)	87 (41)	*χ*^2^ = 5.70; *p* = 0.22
12 years of education	51 (57)	26 (29)	13 (14)	90 (42)	
≥ 3 years of higher education	17 (46)	13 (35)	7 (19)	37 (17)	
Employment (*n*; %) (*n* = 234)					
School or studies	16 (48)	13 (39)	4 (12)	33 (14)	*χ*^2^ = 1.12; *p* = 0.98
Employed	27 (54)	16 (32)	7 (14)	50 (21)	
Not in employment or education	77 (51)	54 (36)	20 (13)	151 (65)	
Current use of substances (*n*; %) (*n* = 215)					
No	72 (49)	52 (36)	22 (15)	146 (68)	*χ*^2^ = 1.70; *p* = 0.43
Yes	36 (52)	27 (39)	6 (9)	69 (32)	
Urgency of referral (*n*; %)					
Elective	24 (35)^*^	35 (51)^*^	10 (14)	69 (29)	*χ*^2^ = 12.45; *p* = 0.002
Acute	99 (59)^*^	48 (28)^*^	22 (13)	169 (71)	
Severity at first contact (*n*; %)					
Outpatient	26 (39)^**^	26 (39)	15 (22)^**^	67 (28)	*χ*^2^ = 13.74; *p* = 0.008
Voluntary inpatient	37 (49)	32 (42)	7 (9)	76 (32)	
Involuntary inpatient	60 (63)^*^	25 (26)^**^	10 (11)	95 (40)	
Final psychosis diagnosis (*n*; %)					
Acute psychosis	32 (68)^**^	4 (9)^*^	11 (23)^**^	47 (20)	*χ*^2^ = 35.42; *p* ≤ 0.001
Schizophrenia or schizoaffective	67 (51)	58 (44)^*^	6 (5)^*^	131 (55)	
Delusional disorders	10 (36)	12 (43)	6 (21)	28 (12)	
Unspecified psychosis	14 (44)	9 (28)	9 (28)^**^	32 (13)	

^†^Flupentixol (*n* = 2), amisulpride (*n* = 2), zuclopenthixol (*n* = 1), prochlorpenthixol (*n* = 1), and ziprasidone (*n* = 1).

^‡^*Post hoc* tests with Bonferroni correction show these groups to be statistically significant in age.

^*^Post hoc tests with Bonferroni correction show these groups contribute to statistical significance.

^**^Post hoc tests without Bonferroni correction show these groups to contribute to statistical significance.

### Antipsychotic medication at illness presentation

**Table 1** shows a comparison of selected variables between the three medication groups: HWPA, L/MWPA, and no antipsychotic medication. A total of 123 (52%) patients were given HWPA, and 83 (35%) patients were given L/MWPA, while 32 patients (13%) received no antipsychotic medication. At initial treatment, the only HWPA prescribed was olanzapine, while quetiapine and aripiprazole were the most commonly used L/MWPA. See **Table 1** for details of other antipsychotics used.

There was a significant difference in median age between the medication groups (*p* = 0.03), and *post hoc* testing showed that the difference between the HWPA group (29 years) and the L/MWPA group (26 years) was significant. There was no significant difference in the prescription of HWPA between female and male patients. There was no significant difference in the prescription of HWPA according to education, employment status, or current substance use at referral.

We found a significant difference in the use of HWPA according to urgency (acute/elective) at referral. Patients referred acutely were more frequently given HWPA (59% of the patients) compared with patients referred electively, where 35% received HWPA (*p* = 0.002).

When stratifying patients based on the severity of the disorder, there was a higher likelihood of receiving HWPA medication with more severe symptomatology/functional difficulties, as reflected in the level of care at referral. In patients with the most severe illness presentation (involuntary inpatient treatment), 63% used HWPA at initiation of treatment, and in patients with the least severe illness presentation (outpatient treatment), 39% used HWPA (*p* = 0.008). This corresponds to a 60% increased likelihood of being prescribed HWPA in the most severely ill patients compared with the least severely ill patients. There was also a low use of L/MWPA in patients with the most severe illness presentation and a relatively high rate of no use of antipsychotics in patients with the least severe illness presentation (see **Table 1**). There was an association between final diagnosis and the antipsychotic medication group patients received as their first treatment (*p* ≤ 0.001). Patients with acute psychosis were more often given HWPA, received less L/MWPA, and had a higher proportion of no antipsychotic medication use. Patients diagnosed with schizophrenia or schizoaffective disorder were more often prescribed L/MWPA, and less frequently treated without antipsychotic medication.

### Use of antipsychotic medication during the 2-year follow-up

#### Chronic psychotic disorders

**Figure 2:**
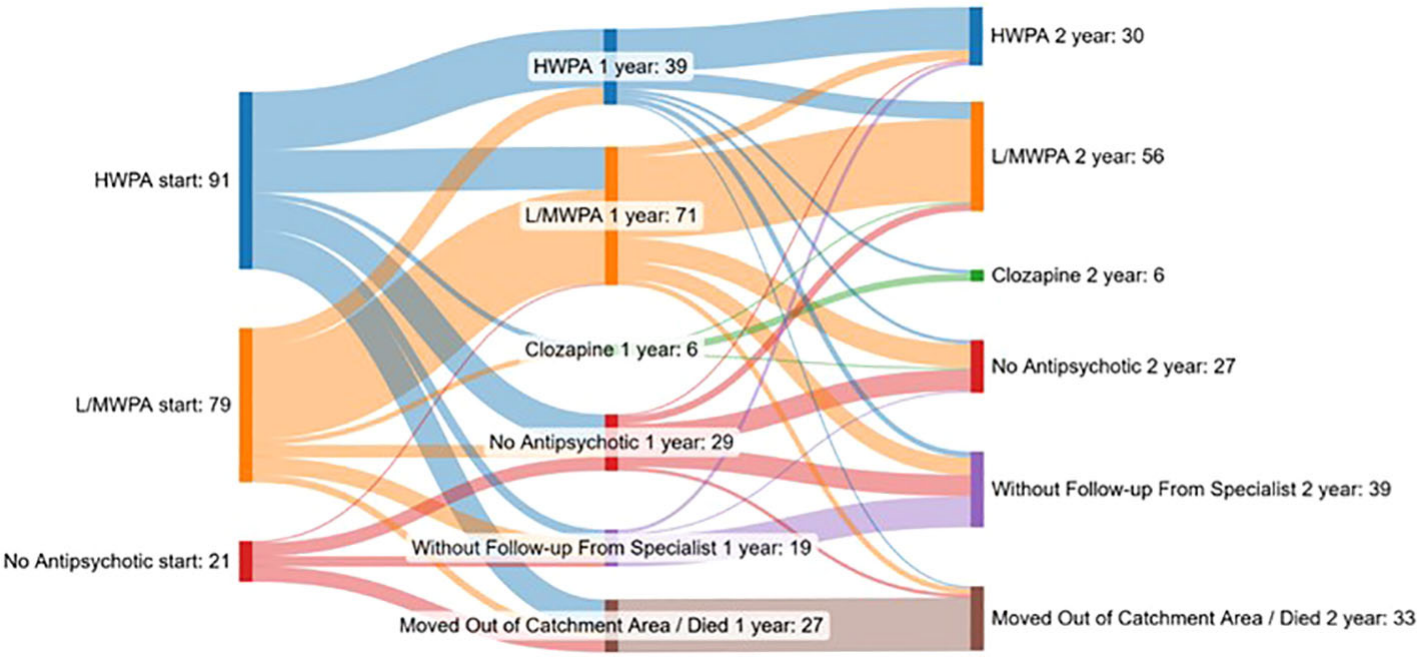
Antipsychotic medication at initiation, 1- and 2-year follow-up in patients with a chronic psychosis diagnosis. HWPA, high weight-inducing potential antipsychotic; L/MWPA, low/medium weight-inducing potential antipsychotic.

At initiation of medication, 47% of patients diagnosed with a chronic psychotic disorder (F20.x, F22.x, F25.x, and F29) used HWPA, and 41% used L/MWPA. **Figure 2** shows the flow between medication groups from initiation through the 1- and 2-year follow-up. The rates of patients using HWPA and L/MWPA were 27% vs. 49% and 25% vs. 47% at the 1- and 2-year follow-up, respectively, among patients for whom follow-up data were available.

The proportion of patients continuing with the same medication group as they initiated was almost twice as high among patients starting with L/MWPA (61%) compared with those starting with HWPA (33%) at the 1-year follow-up. At the 1-year follow-up, 60% of patients initiating HWPA were using antipsychotic medication of any type, while the corresponding rate in the L/MWPA group was 76%. When comparing the continuation rates at the 1-year follow-up for the three most commonly used antipsychotics at initiation, olanzapine was continued in 33% of patients, aripiprazole in 39%, and quetiapine in 26% of patients originally initiated on that medication.

See **Figure 1** for shifts in the use of antipsychotics from the 1- to 2-year follow-up. Clozapine was used at both the 1- and 2-year follow-up by six patients; in total, eight patients used or had used clozapine during the 2-year follow-up, corresponding to 6% to 7% of the patients for whom we had follow-up data at the 1- and 2-year follow-up.

#### Acute psychotic disorders

**Figure 3:**
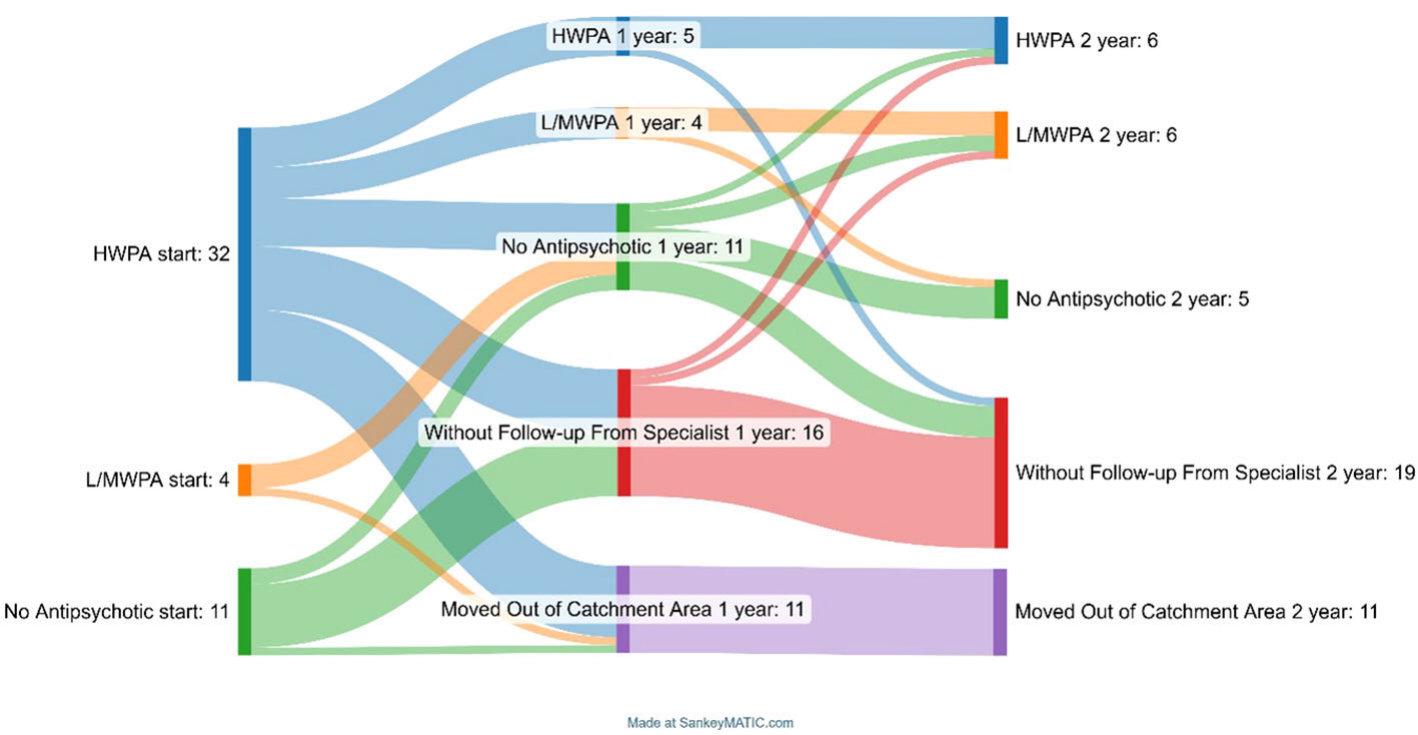
Antipsychotic medication at initiation, 1- and 2-year follow-up in patients with an acute psychosis diagnosis. HWPA, high weight-inducing potential antipsychotic; L/MWPA, low/medium weight-inducing potential antipsychotic.

HWPA was used as the first antipsychotic treatment in 68% of patients with an acute psychotic disorder and accounted for 89% of initial antipsychotic medication. **Figure 3** describes the flow between groups at initiation and at the 1- and 2-year follow-up. A large proportion of patients (57% and 64%) did not have follow-up from specialist mental healthcare after 1 and 2 years, respectively. Between 20% and 25% of patients were still using antipsychotic medication at the 1- and 2-year follow-up, and three of the 12 patients using antipsychotic medication at the 2-year follow-up were diagnosed with bipolar disorder during the follow-up period.

## Discussion

The main findings in this study were that a large proportion (52%) of patients were initiated with HWPA as their first antipsychotic medication, with olanzapine being the only HWPA used. L/MWPA were prescribed to 35% of patients, and 13% did not receive any antipsychotic medication at illness presentation. Older age, acute referral, and greater severity of psychotic condition at illness presentation increased the likelihood of treatment with HWPA. Among patients with chronic psychotic disorders, approximately twice as many who started with L/MWPA continued with the same group of medication at 1-year follow-up compared with those who started with HWPA (61% vs. 33%). During the first year of follow-up, the proportion of patients with chronic psychotic disorders using HWPA decreased from 47% to 27%. This proportion remained more stable during the second year of follow-up, decreasing only from 27% to 25%.

Our finding of 52% of the patients being prescribed HWPA as the first choice of antipsychotic medication is comparable to findings from a cohort of patients with first-episode affective and nonaffective psychosis recruited in Denmark and Norway from 1997 to 2000, in which HWPA was used in 48% of patients (33). Another Norwegian study of patients with first-episode schizophrenia in the Stavanger region found HWPA to be used in 44% as the first choice of antipsychotic medication (26). Both studies were clinical research projects with inclusion rates ranging from 64% to 74% of eligible patients and were conducted from 1997 to 2000 (33) and 2002 to 2013 (26), respectively. In contrast, our study included all patients in the catchment area diagnosed with FEP and their first prescribed antipsychotic medication were included, thereby providing more representative data for the FEP population and from a more recent period.

In contrast to these findings, a Norwegian register-based population study including all patients with a FEP in Norway found relatively low use of HWPA, with only 19% of the population using medication from this group and only 54% using antipsychotic medication in total (34). This study included patients from 2012 to 2019, overlapping in time with our study. However, a register-based study has several limitations, as discussed by the authors, including the underestimation of the use of HWPA and antipsychotics in general when medications used by inpatients were not included and the overestimation of the frequency of FEP when a single registered contact from the Norwegian Patient Registry was used, which may consequently underestimate the use of antipsychotics in general and HWPA.

Findings from other countries show large variations in the use of HWPA as the first prescribed antipsychotic medication. Numbers in cohorts similar to ours range from 13% to 81%, with the lowest rates reported in studies from the USA, Spain, and the UK (18, 19, 22), medium rates reported in a study from the UK (23), and the highest rates reported in a study from Ireland (21). These large variations in the use of HWPA as the first choice of antipsychotic medication are not explained by differences in prescription rates over time, as the inclusion periods of studies with low, medium, and high rates of HWPA use overlap, ranging from 2005 to 2017.

In studies from Norway, the rates of HWPA seem to be stable from 1997 to 2013, and in the register-based study from Norway, despite the low use of HWPA, the use of olanzapine remained stable during the inclusion period from 2012 to 2019 (34). Therefore, the large variations in the use of HWPA medication may be due to differences in clinical practice. Findings of differences in prescription rates across different geographical regions within the same country have been reported in both the USA (19) and the UK (18, 23).

We found that higher age, acute referral, more severe illness at referral, and a diagnosis of an acute psychotic disorder increased the likelihood of HWPA initiation as the first antipsychotic medication. One other study found a similar association between the severity of the disorder and the use of olanzapine. Keating et al. found that a higher functioning assessed with GAF decreased the risk of olanzapine being used as the first-choice medication in FEP (21). They did not find age or diagnostic category to significantly influence whether olanzapine or another antipsychotic medication was used.

One possible reason for the widespread use of olanzapine may be that it is regarded as more effective (35) and has side effects, such as sedation, that reduce the need for the use of benzodiazepines or hypnotic medication, especially during the acute stages of psychosis.

For patients with a chronic psychotic disorder, the continuation rate of the same medication group at the 1-year follow-up was twice as high among patients initiating with L/MWPA compared to those initiating with HWPA. This could indicate that more patients were satisfied with treatment in the L/MWPA group and were more willing to continue using antipsychotic medication. A recent meta-analysis found an increased likelihood of nonadherence or discontinuation in patients using olanzapine compared to those using antipsychotics with a lower probability of weight gain (36). However, it must be acknowledged that any switch in medication due to inadequate effect or side effects in the HWPA group would lead to a shift to the L/MWPA group, as there are no other SGA antipsychotics in the HWPA group. Therefore, the comparison between the numbers continuing in their initial group of antipsychotics is not without limitations. Although a higher proportion of patients initiating L/MWPA used any antipsychotic medication at the 1-year follow-up compared to patients initiating HWPA, differences in several patient characteristics at referral may explain differences in treatment discontinuation at the 1-year follow-up. We did not have the possibility to control for such characteristics in this study.

The 1-year discontinuation rate of the first prescribed antipsychotic medication is generally reported to range from 50% to 70% (20, 37–39) and appears to be lower in clinical studies (39) compared to naturalistic studies (20, 37, 38). These discontinuation rates are in line with those found for the three most frequently used antipsychotics in our study.

Regarding the use of clozapine, few patients were treated with clozapine during the 2-year follow-up (6%–7%). This is comparable to other cohorts both from Norway and internationally: 4% in a Norwegian study with 1-year follow-up (26); 7%–8% in two Spanish studies (22, 40) with 2-year follow-up; and 15% in a UK study with 5-year follow-up (41).

For patients with an acute psychotic disorder, 89% initiated antipsychotic medication with HWPA. Given the nature of acute psychotic disorders, it is natural that many of the patients did not receive treatment from specialist mental healthcare at the 1- and 2-year follow-up. Even if medication is used for a shorter time period than for patients with a chronic psychotic disorder, the risk of weight gain remains high, considering the findings of significant weight gain during the first 13 weeks of treatment (11). Of note, 25% of the patients diagnosed with acute psychosis were using antipsychotic medication after 2 years. For a minority, a bipolar disorder was diagnosed, which is a valid reason for the use of antipsychotic medication, but for the majority, more chronic illness trajectories may be suspected, although this was not formally diagnosed.

Several modern guidelines for the treatment of FEP now recommend specific medications as first-choice treatment in FEP (14, 16, 17) and, together with other guidelines, recommend not using HWPA as first-line treatment (3, 42). The recommendation not to use HWPA is also included in the new guidelines in Norway, published in 2025 (24). The findings regarding demographic and clinical associations with high HWPA medication use indicate a need for focused attention on antipsychotic prescribing practices in specific patient groups or hospital units. However, the relatively high rates of HWPA as the first antipsychotic in this population require a broader focus on this practice. However, HWPA prescriptions do not necessarily reflect nonadherence to guidelines or suboptimal care. It can also reflect compensatory prescribing practice, where patients at high risk of weight gain are given antipsychotics other than HWPA.

The generalizability of our findings to current practice in Norway is supported by a 7% increase in the use of olanzapine from 2016 to 2020 and by the fact that other regional health trusts in Norway have higher prescription rates than St. Olavs University Hospital; the latest available data from the Norwegian Prescription Database (NorPD) are from 2020 (43).

The main strength of this study is the inclusion of all FEP patients in a defined catchment area, where there is only one hospital providing acute and inpatient treatment for mental health disorders. We have data on the first antipsychotic medication initiated in all FEP patients who started with medication. The naturalistic setting of the study provides representative data on antipsychotic medication used in a FEP population, including not only patients who consent to research projects or collect prescribed medication from pharmacies. The naturalistic setting also leads to limitations, as we only have data on patients who are still in treatment at the 1- and 2-year follow-up. Another limitation is that the data used represent clinical practice 8 to 12 years ago; more recent data do not show any changes in rates of HWPA use in this population (34), and national prescription levels of olanzapine are increasing (43), so the findings are likely representative of current practice. Furthermore, it is a limitation that the diagnoses are based on routine clinical investigations and not necessarily based on structured clinical interviews. The retrospective nature of the study also limits the possibility of including more extensive clinical data that are not reliably recorded in patient charts.

## Conclusion

A large proportion (52%) of patients were initiated with a HWPA as the first choice of medication. Higher age, acute referral, severity of illness, and an acute psychotic disorder were associated with a higher risk of HWPA medication. For patients with a chronic psychotic disorder, the continuation rates for L/MWPA were twice as high as those for HWPA in the first year. Acute psychotic disorders had a higher frequency of initiation with HWPA than chronic psychotic disorders, but also a shorter treatment duration. National and international guidelines advise against using HWPA as the first choice in FEP treatment. Significant efforts are needed to align clinical practice more closely with guideline recommendations.

## Author's note

This article was originally written as a graduate thesis in medicine by ML, under the guidance of MS and GM.

## Data Availability

The raw data supporting the conclusions of this article will be made available by the authors, without undue reservation.
